# Adaptive NK Cell Therapy Modulated by Anti-PD-1 Antibody in Gastric Cancer Model

**DOI:** 10.3389/fphar.2021.733075

**Published:** 2021-09-13

**Authors:** Shahrokh Abdolahi, Zeinab Ghazvinian, Samad Muhammadnejad, Mohammad Ahmadvand, Hamid Asadzadeh Aghdaei, Somayeh Ebrahimi-Barough, Jafar Ai, Mohammad Reza Zali, Javad Verdi, Kaveh Baghaei

**Affiliations:** ^1^ Department of Applied Cell Sciences, School of Advanced Technologies in Medicine, Tehran University of Medical Sciences, Tehran, Iran; ^2^ Cell-Based Therapies Research Center, Digestive Diseases Research Institute, Tehran University of Medical Sciences, Tehran, Iran; ^3^ Hematology-Oncology and Stem Cell Transplantation Research Center, Tehran University of Medical Science, Tehran, Iran; ^4^ Department of Hematology and Applied Cell Sciences, Faculty of Medical Sciences, Tarbiat Modares University, Tehran, Iran; ^5^ Basic and Molecular Epidemiology of Gastrointestinal Disorders Research Center, Research Institute for Gastroenterology and Liver Diseases, Shahid Beheshti University of Medical Sciences, Tehran, Iran; ^6^ Department of Tissue Engineering, School of Advanced Technologies in Medicine, Tehran University of Medical Sciences, Tehran, Iran; ^7^ Gastrointestinal Disorders Research Center, Research Institute for Gastroenterology and Liver Diseases, Shahid Beheshti University of Medical Sciences, Tehran, Iran

**Keywords:** anti-PD-1 antibody, NK cell therapy, immune checkpoint inhibitor, immunotherapy, gastric cancer

## Abstract

Recently, adaptive NK cell therapy has become a promising treatment but has limited efficacy as a monotherapy. The identification of immune checkpoint inhibitor (ICI) molecules has opened a new horizon of immunotherapy. Herein, we aimed to demonstrate the cytotoxic effects of a polytherapy consisting of *ex vivo* expanded IL-2-activated NK cells combined with human anti-PD-1 antibody as an important checkpoint molecule in a xenograft gastric cancer mouse model. EBV-LCL cell is used as a feeder to promote NK cell proliferation with a purity of 93.4%. Mice (NOG, female, 6–8 weeks old) with xenograft gastric tumors were treated with PBS, *ex vivo* IL-2-activated NK cells, IL-2-activated NK cell along with human anti-PD-1 (Nivolumab), and IL-2-activated pretreated NK cells with anti-PD-1 antibody. The cytotoxicity of *ex vivo* expanded NK cells against MKN-45 cells was assessed by a lactate dehydrogenase (LDH) assay. Tumor volume was evaluated for morphometric properties, and tumor-infiltrating NK cells were assessed by immunohistochemistry (IHC) and quantified by flow cytometry. Pathologic responses were considered by H and E staining. *Ex vivo* LDH evaluation showed the cytotoxic potential of treated NK cells against gastric cancer cell line. We indicated that the adoptive transfer of *ex vivo* IL-2-activated NK cells combined with anti-PD-1 resulted in tumor growth inhibition in a xenograft gastric cancer model. Mitotic count was significantly decreased (*p < 0.05), and the tumor was associated with improved infiltration of NK cells in the NK-anti-PD-1 pretreated group (*p < 0.05). In conclusion, the combination approach of activated NK cells and anti-PD-1 therapy results in tumor growth inhibition, accompanied by tumor immune cell infiltration in the gastric tumor model.

## Introduction

Gastric cancer (GC) is the fifth leading cause of cancer and the third leading cause of death. (Thrift and El-Serag 2020). GC has low clinical symptoms, and it usually progresses at the time of diagnosis, making it challenging to treat patients. Various treatments can be considered, including surgery, chemotherapy, radiation therapy, and targeted therapies (Meza-Junco, Au et al., 2011; Wadhwa, Taketa et al., 2013). However, even after conventional treatments, many patients experience a recurrence of the disease and eventually, cancer metastasizes to other tissues (Enzinger and Mayer 2003). Therefore, consideration of innovative therapeutic approaches is of great importance. Indeed, there is an increasing investigation in establishing an effective immune cell-based therapy for GC by *ex vivo* activating and expanding immune cells. Multifarious studies have presented the therapeutic potential of effective immunotherapy of immune cells (Rezvani, Daher et al., 2020; Ingram, Madan et al., 2021).

Natural killer (NK) cells are promising approaches in treating solid tumors that recognize and lyse infected and malignant cells and exert their cytotoxicity effect without prior sensitization (Close 2016; Jung et al., 2018). NK cells are stimulated as anticancer agents by downregulating or lost MHC-I molecules, a process in which tumor cells can usually escape from cytotoxic T lymphocytes (CTLs) recognition (van Erp, van Kampen et al., 2019). Furthermore, NK cells activation is related to the balance between activating and inhibitor receptors and independent of antigen-presenting cells (APC) (Ljunggren and Malmberg 2007). Despite the advantages of NK therapy, there are major challenges in tumor infiltration or tumor site suppression (Li, Zhang et al., 2016; Melaiu, Lucarini et al., 2020). In an immunological response context, a tumor without infiltrating lymphocytes (TILs) is defined as a “non-inflamed” or “cold” tumor (Herbst, Soria et al., 2014; Mlecnik, Bindea et al., 2016). In contrast, “hot” tumors show a high number of TILs, making the TME more responsive to immunotherapeutic interventions (Kitano, Ono et al., 2017).

There are reasons for the tumor site suppression of adaptive NK cell monotherapy, including (i) myeloid-derived suppressor cells (MDSCs) and Tregs function (Pedroza-Pacheco, Shah et al., 2016; Liu, Wei et al., 2018); (ii) overexpression of MHC class I and MICA/B (Malmberg, Carlsten et al., 2017; Raneros, Puras et al., 2017); (iii) the expression level changes in activating and inhibitory receptors of NK cells (Pietra, Manzini et al., 2012; Davis, Vallera et al., 2017); (iv) marginal infiltration of NK cells (Uong, Lee et al., 2018). Therefore, any approaches to increase the efficacy of NK therapy should address the mentioned limitation. Among them, immune checkpoint inhibitors (ICI) have a crucial role in the cytotoxicity of NK cells.

PD-1 is a surface receptor known as an immunological checkpoint inhibitor for immune cells such as myeloid cells, thymocytes, activated T cells, and NK cells (Nishimura and Honjo 2001; Cheng, Veverka et al., 2013). PD-L1/2 ligands are expressed by various tumor cells, including liver cancer, breast, and GC (Engel, Honig et al., 2014; Jung, Jeong et al., 2017; Wu, Cao et al., 2017). By binding to its ligands, PD-1 plays a vital role in immunosuppressing by exhausting immune cells, increasing Tregs, reducing autoimmunity, and promoting tolerance (Keir, Butte et al., 2008; Francisco, Sage et al., 2010; Fife and Pauken 2011). Thus, blocking this inhibitory pathway is a promising approach to increase the efficacy of cancer immunotherapy (Topalian, Drake et al., 2012). These therapies can be well-tolerated compared with chemotherapy and provide long-term survival (Chiossone, Vienne et al., 2017). In addition, the PD-1 receptor can be targeted by a fully humanized IgG4 antibody (Nivolumab) and can attenuate the inhibitory signal of NK and T, responding to treatment in highly immunogenic tumors (Li, Shao et al., 2018; Havel, Chowell et al., 2019; Desai, Deva et al., 2020).

There have been limited studies on adaptive NK therapy on GC; however, it has been demonstrated that the infiltration and cytotoxicity effects of NK are major problems (Chen, Yang et al., 2014; Du and Wei 2019). Therefore, in the current study, to overcome the limitations, we surveyed the combination therapy of activated NK cells with Nivolumab as an anti-PD-1 inhibitor to enhance tumor infiltration and cytotoxicity of NK against gastric cancer tumors.

## Materials and Methods

### Cell Lines and Culture Media

The MKN-45 cell line (human gastric cancer cell line) was purchased from the Iranian Biological Resource Center (Tehran, Iran). Epstein-Barr virus (EBV)–transformed lymphoblastoid cell line (LCL) as feeder cells was maintained in Roswell Park Memorial Institute-1640 (RPMI-1640) (Gibco, United States); media were supplemented with 10% FBS (Gibco, United States), 1% penicillin-streptomycin, and 1% L-glutamine.

### Human NK Cell Isolation, *Ex Vivo* Expansion, and Activation

NK cells were isolated from healthy donors’ buffy coats with Ficoll Paque Premium (GE Healthcare’s, United States) gradient centrifugation. According to the manufacturer’s instructions, NK cells were collected by negative selection using a human NK cell isolation kit (Miltenyi Biotech, Germany). The purity of NK cells was assessed by flow cytometry analysis of CD3^−^ and CD56^+^ markers (FACSCalibur Becton Dickinson, United States). EBV-LCL was applied for optimal NK cell expansion. EBV-LCL cell line was established by culturing peripheral blood mononuclear cells (PBMCs) in the presence of 100 μg/ml cyclosporin A with EBV supernatant harvested from the cell line B95-8 (Iranian Biological Research Center) (Igarashi, Wynberg et al., 2004). The procedure is based on an existing expansion protocol for *in vivo* studies (Berg, Lundqvist et al., 2009) that utilizes 100 Gy-irradiated EBV-LCL as feeder cells (at a ratio of 1:10) trigger NK cell proliferation and highly activated NK cells. In the first 5 days, NK cell colonies are formed, and every 3 days, a fresh medium enriched by IL-2 (500 IU/ml) (Miltenyi Biotech, Germany) is added to the cells for 21 days.

### Cytotoxicity Assays

The cytotoxic effects of activated NK cells as an effector cell (E), whether alone and in combination with anti-PD-1, were defined in a co-culture of MKN-45 as a target cell (T). The ratios of 1:1, 1:3, and 1:6 were studied. LDH assay was performed after 24 h of incubation as a necrosis marker in a cell culture medium (Chan, Moriwaki et al., 2013).

### Heterotopic Gastric Cancer Mouse Model

All animals were housed in individually ventilated cages (IVC) for animal experiments. The center maintains standardized housing conditions and hygiene management according to the guidelines of the ethics committee. The animals are preserved under special conditions, e.g., a 12/12 h light-dark cycle, a humidity of 65%, and a temperature of 25°C. To assess the antitumor effect of NK cells in combination with Nivolumab (Bristol Myers Squibb, USA) *in vivo*, we used 6–8-week-old female NOD. Cg-Prkdcscid IL2rgtm1Sug (NOG) mice were obtained from the animal facility of the Digestive Disease Research Institute of Tehran University of Medical Sciences, which subcutaneously inoculated by 5×10^6^ MKN-45 cells. Prior to euthanasia, animals are anesthetized with ketamine and xylazine and euthanized with CO_2_. Tumor sizes and body weights were measured twice a week. Animals with a tumor volume ≥2000 mm^3^, real bodyweight loss ≥20%, or BC = 1 (body condition) were humanely terminated. All animal experiments were authorized by the institutional ethics committee of the Tehran University of Medical Science (IR.TUMS.MEDICINE.REC.1399.644).

### Sample Size, Dosages, and Schedule of Administration

**FIGURE 1:**
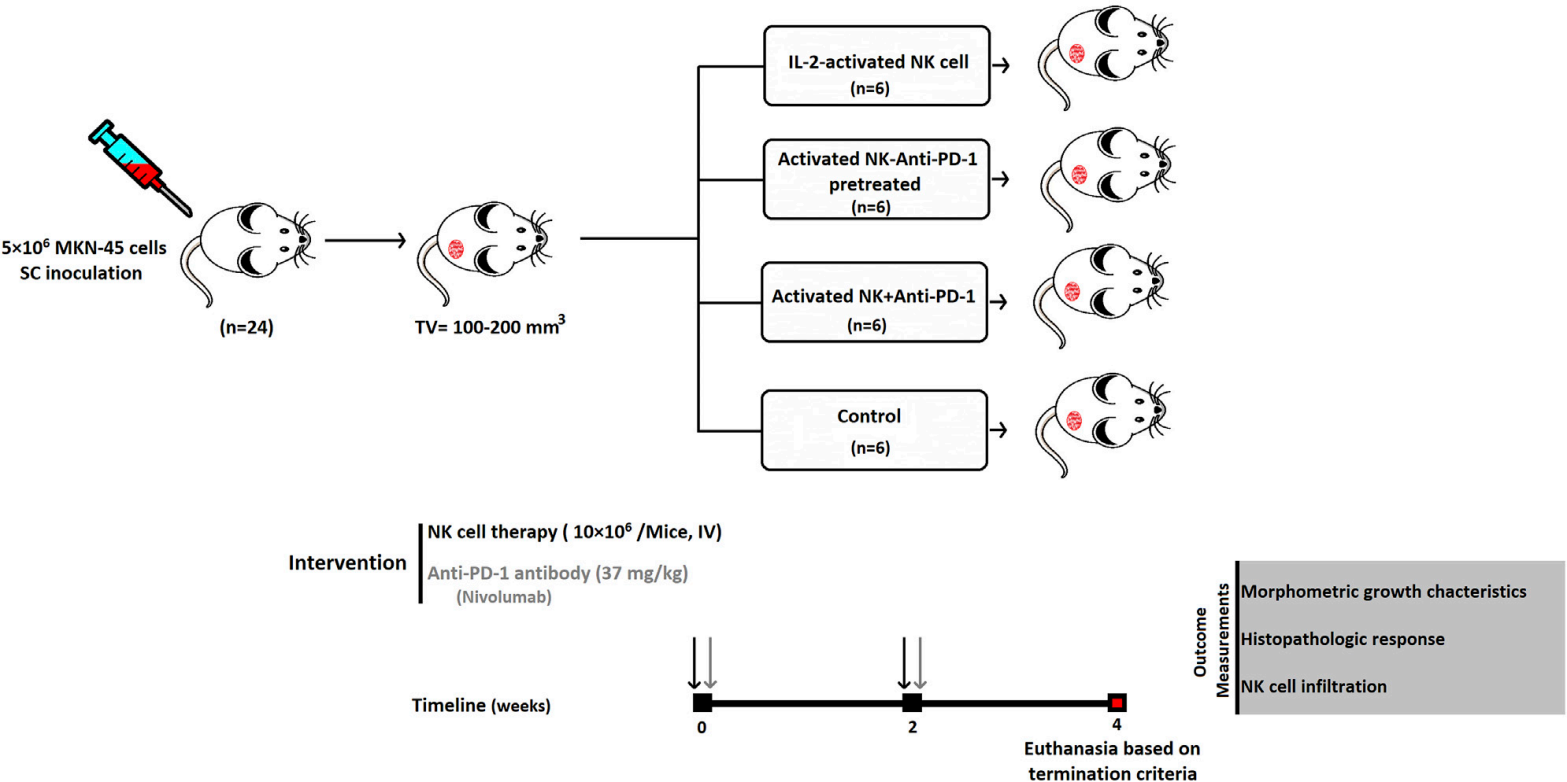
Schematic diagram of anti-PD-1 antibody combined with NK-based immunotherapy in a gastric cancer xenograft model. SC, subcutaneous; IV, intravenous.

Tumor median size of 100–200 mm^3^ (day 0) was selected for dividing the animals randomly into four experimental groups, including IL-2-activated NK cells (NK cells), IL-2-activated NK cells along with anti-PD-1 antibody (Nivolumab) (NK + anti-PD-1), and IL-2-activated NK cells *ex vivo* pretreated with 20 μg/ml anti-PD-1 (NK-anti-PD-1 pretreated). Each mouse received 10×10^6^ NK cells twice at 2-week intervals *via* the IV route (Figure 1). The dosage of anti-PD-1 (Nivolumab) was translated from human into murine setting based on body surface area using the following formula (Nair and Jacob 2016):

$$\text{Murine equivalent dosage}\left(\text{unit}/\text{kg}\right)=\text{ pediatric dosage}\left(\text{unit}/\text{kg}\right)\text{×}\frac{Human\;Km}{Mouse\;Km}.$$

The Km constant was 37 for adult humans 37 and 3 for mice (Reagan-Shaw et al., 2008). The NK + anti-PD-1 group was treated with 37 mg/kg anti-PD-1 antibody twice at 2-week intervals *via* the IV route. Control is the fourth group, which received phosphate-buffered saline (PBS).

### Tumor Morphometric Outcomes

Tumor volume was measured by a caliper and calculated by the following formula: tumor volume = 1/2 (length × width 2). Relative tumor volume (RTV) was evaluated for morphometric growth kinetics of the tumors by dividing tumor volume on a measured day to tumor volume on day 0. Antitumor activity was presented by tumor growth inhibition (TGI) percentage using a formula by Tsukihara et al.: TGI (%) = [1 − (RTV of the treated group)/(RTV of the control group)] × 100 (%) (Tsukihara, Nakagawa et al., 2015).

### Flow Cytometry

NK cells characterization by PE anti-human CD56^+^ and FITC anti-human CD3^−^ markers was quantitatively evaluated; the infiltration of NK cells at the tumor site was performed using flow cytometry (FACSCalibur Becton Dickinson, United States). NKG2D and CD69 were evaluated before and after treatment of IL-2-stimulated NK cells by the anti-PD-1 antibody for further characterization. The whole tumor was collected and digested mechanically and enzymatically, and staining was done according to the manufacturer's instructions; then, flow cytometry was performed (Feng, Peng et al., 2009). Data were analyzed using the FlowJo 7 software (Tree Star, Inc.). Experiments were carried out in triplicate and quantified by GraphPad Prism 9 (GraphPad Software Inc., CA, United States).

### Histopathology Assessments

The histopathologic response of tumors to the interventions was assessed based on the residual tumor (R) (Edge, Byrd et al., 2010) (Table 1). Hematoxylin and Eosin (H and E) stain slides were prepared to stratify the histopathologic responses. Proliferative activity changes assessed mitotic count on H and E slides. As previously described by Meuten and colleagues, mitotic cells are counted in areas and reported as an average over ten consecutive high power fields (HPF) (Meuten, Moore et al., 2016).

**TABLE 1:** Residual tumor (R) classification.

Classification	Description
R0	The entire tumor is destroyed in response to treatment
R1	More than 70% of the tumor is destroyed in response to treatment, and fibrosis and diffuse apoptosis (within the tumor) are observed
R2	30 and 70% of tumors in response to treatment have undergone fibrosis and apoptosis
R3	The response rate to treatment is very low or non-existent

Immunohistochemistry (IHC) against human CD56 (Biolegend, United States) was performed as described previously (Kim et al., 2016) for NK cell infiltration evaluation. Furthermore, the active protein of caspase 3 for apoptosis induction evaluation was detected using a rabbit polyclonal antibody (Biolegend, United States). IHC was assessed based on the Allred score; this scoring combines the percentage of immunoreactive cells and the intensity score (Parvin et al., 2019) (Table 2). Following the euthanasia of the mice, tumor tissues were collected and processed for paraffin embedding. Briefly, paraffin-embedded tumors were cut by microtome to a thickness of 4 μm. After deparaffinization and hydration, the tumor tissues were incubated by primary and secondary antibodies (Adan, Alizada et al., 2017).

**TABLE 2:** The Allred score combined intensity and percent of immunoreactive cells.

Positive cells %	Proportion score		Intensity	Intensity score
No cells are immunoreactive	0		Negative	0
≤1%	1		Weak	1
1–10%	2		Intermediate	2
11–33%	3		Strong	3
34–66% o	4			
67–100%	5			
**Aggregation of proportion and Intensity score**				
0–1		Negative		
2–3		Weak positive		
4–6		Intermediate positive		
7–8		High positive		

### Statistical Analysis

All results are reported as the mean ± SEM and the data were analyzed by GraphPad Prism 9 software package (GraphPad Software, Inc., San Diego, United States). A *p* value of <0.05 represented statistical significance.

## Results

### *Ex Vivo* Expansion and Immunophenotyping of NK Cells

**FIGURE 2:**
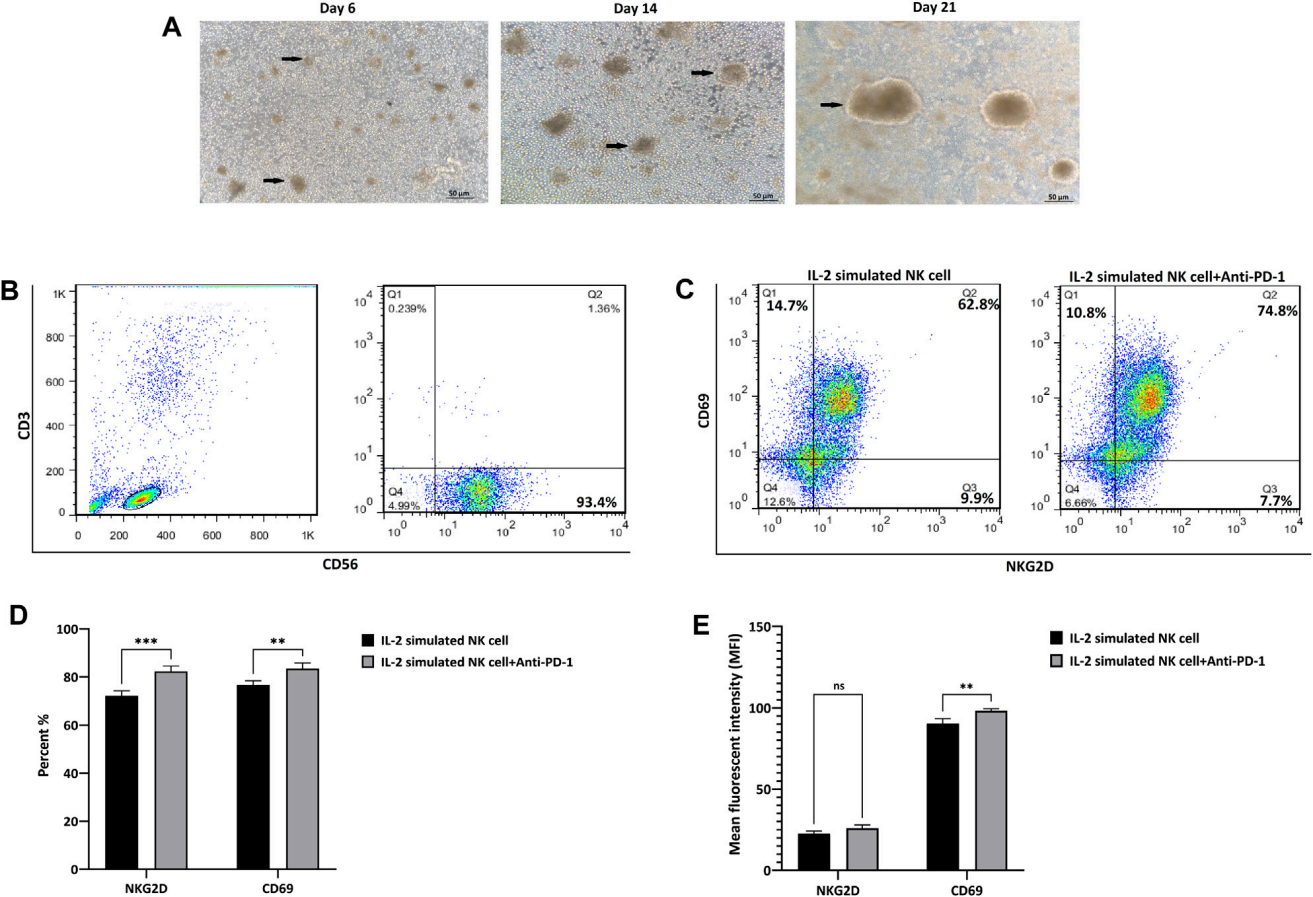
Isolation, characterization*,* and expansion of NK cells. **(A)** The morphology of NK cells after 6, 14, and 21 days of round-shaped colony of NK cells is illustrated by arrows. **(B)** The frequency of CD56+CD3-NK cells in PBMC-derived NK cells, after MACS isolation with more than 93.4% purity. **(C)** The frequency of NKG2D and CD69 marker before and after treatment of IL-2-stimulated NK cells by anti-PD-1 antibody. **(D)** The quantification of positive cells for NKG2D and CD69 marker. **(E)** The quantification of mean fluorescence intensity for NKG2D and CD69 marker. Data presented as means ± standard error (M ± SE) for three independent experiments. Statistical significance was determined using one-way ANOVA with *p < 0.05. Scale bar: 50 µm, magnification 200X. ns, not significant.

NK cells isolated from peripheral blood were characterized by the presence of CD56 and the absence of CD3 as NK cell surface biomarkers. The purity was 93.4%, which included the CD3^−^ CD56^+^ cells based on flow cytometric analysis. Freshly isolated CD3- and CD56+ cells were cultured in the presence of IL-2 and feeder cells and expanded for 21 days. Expanded NK cells exhibited clonal growth and round shape morphology under cytokine-/feeder-enriched (IL-2/EBV-LCL cells) medium; finally, after 21 days, we reached 370 million cells with more than 95% viability, which were evaluated by trypan blue before injection (Figures 2A,B).

Further investigation of NK + anti-PD-1 immunophenotyping NKG2D and CD69 was performed using flow cytometry. NK NKG2D^+^ and CD69^+^ cells increased 10 and 8 percent after treatment with IL-2-stimulated NK cells with anti-PD-1 antibody (*p* < 0.0007 and *p* < 0.0085, respectively). Moreover, quantification of mean fluorescence intensity (MFI) showed that anti-PD-1 treatment improved CD69 expression (*p* < 0.002), but no significant changes were observed in NKG2D marker after treatment with IL-2-stimulated NK cells with anti-PD-1 (Figures 2C,D,E).

### *Ex Vivo* Cytotoxicity of IL-2-Activated NK Cells Combined With Anti-PD-1

**FIGURE 3:**
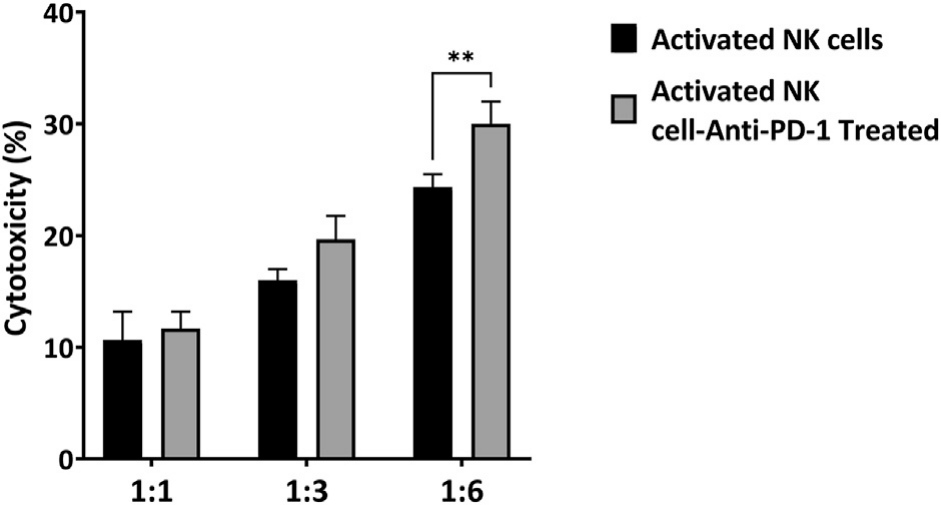
Cytotoxicity of *ex vivo* activated NK cells and anti-PD1 antibody-treated NK cells toward MKN-45 cell at different E: T ratios. Data presented as means ± standard error (M ± SE) for three independent experiments. Statistical significance was determined using one-way ANOVA with *p < 0.05.

To evaluate the effect of a combined strategy of IL-2-activated NK cells with anti-PD-1 antibody *in vitro,* MKN-45 cells were co-cultured with activated NK cells at three specific E: T ratios (1:1, 3:1, and 6:1). After a 24 h incubation, the NK-cell-mediated cytotoxicity against MKN-45 was detected by LDH assay. In the non-treated group, NK cells showed 11, 16, and 25 percent cytotoxicity at the following E: T ratios: 1:1, 3:1, and 6:1, respectively. In the anti-PD-1-treated group, the lysis percentages were 13, 19, and 30. The most prominent antitumor cytotoxicity of activated NK cells was achieved when PD-1 was inhibited with an anti-PD-1 antibody (*p* < 0.006) (Figure 3).

### The Therapeutic Effect of NK Cells Based on Morphometric Growth Characteristics in Gastric Cancer Xenograft Model

In order to explore the effect of adaptive NK cell therapy combined with Nivolumab in tumor growth *in vivo*, we further investigated the impact of interventions on the morphometric properties in the subcutaneous transplantation mouse model of gastric cancer using MKN-45 cell line.

**FIGURE 4:**
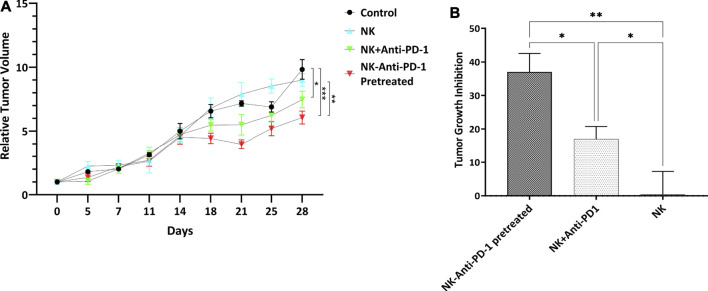
Morphometric growth characteristics. **(A)** RTV versus the elapsed time curve showed tumor growth variations. **(B)** Tumor growth inhibition of interventions normalized with control tumor. Data presented as means ± standard error (M ± SE). Statistical significance was determined using 2-way ANOVA with *p < 0.05.

The use of an Anti-PD-1 antibody confirms the hypothesis that PD-1/PDL-1 pathway inhibition can modulate the therapeutic cytotoxicity effect of NK cells. Ten days after cell line inoculation, tumors reached the desired size of 100–200 mm^3^ for interventions. The sham-treated mice showed the most rapid tumor growth. Two IV injections of *ex vivo* IL-2-activated NK cells combined with an anti-PD-1 antibody caused a significant tumor growth delay. The morphometric growth curves of tumors are shown in Figure 4A. Optimal percent of TGI was observed 28 days after the beginning of treatment. The highest tumor growth inhibition was considered in the NK-anti-PD-1 pretreated group, with a median TGI of 38% (31–41). Also, the NK + anti-PD-1 therapy group showed an inhibitory effect with a median TGI of 18% (12–19); NK cells as monotherapy did not show an inhibitory effect on the gastric cancer model with a median TGI of -0.5%. NK-anti-PD-1 pretreated group significantly inhibited tumor growth more than NK + anti-PD-1 and NK cells (*p* < 0.01 and *p* < 0.001, respectively). Furthermore, NK + anti-PD-1 induces tumor inhibition compared to NK cells (*p* < 0.03). The best therapeutic outcome was observed after modulating NK cells by anti-PD-1 antibody (Figure 4B).

### Effects of Experimental Interventions on Histopathologic Outcomes

PD-1 blockade was used to improve response to the immune-cell-based therapy approach. After implantation of 5×10^6^ MKN-45 cells, the mice bearing tumor was intravenously injected with 10×10^6^ NK cells, NK-anti-PD-1 pretreated, NK + anti-PD-1, and PBS (control).

**FIGURE 5:**
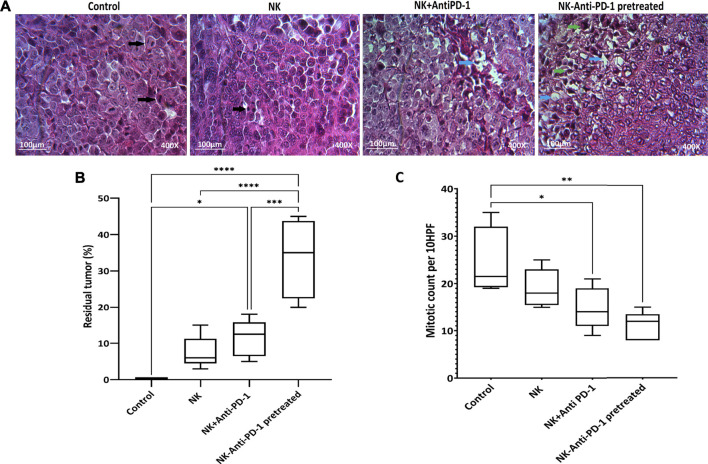
Histopathological finding. **(A)** Representative Hematoxylin and Eosin (H and E). Black arrows showed mitosis, blue arrows showed fibrotic areas, and green arrows showed apoptotic bodies. **(B)** Boxplots showed tumor response based on the residual tumor. **(C)** Mitotic count as proliferation score assessed in 10HPF; anaphase cell division stage is visible in the control tissue sample. Data presented as means ± standard error (M ± SE). Statistical significance was determined using one-way ANOVA with *p < 0.05. Scale bar: 100 µm, 400X magnification.

NK-anti-PD-1 pretreated group based on residual tumor showed that in more than 30% of tumors, the fibrosis areas and apoptotic fragment were increased, R2 histologic response (*p* < 0.0001), in response to intervention. Furthermore, the NK + anti-PD-1 group showed a low response rate, R3 histologic response (*p* < 0.02). NK cells showed a degree of histopathologic response, which not statistically significant (Figures 5A,B).

Mitotic count was investigated to evaluate the effect of interventions on tumor proliferation intensity. NK cells anti-PD-1 pretreated showed the highest anti-proliferative effect; the mitotic count in this group was significantly lower than that in the control group (12 [8–15] vs. 21.5 [19–35] (*p* < 0.002). Furthermore, the mitotic count of the NK + anti-PD-1 group was significantly decreased compared to the control 14 [9–21] (*p* < 0.03), but NK cells did not show a difference in mitotic count (Figure 5C).

**FIGURE 6:**
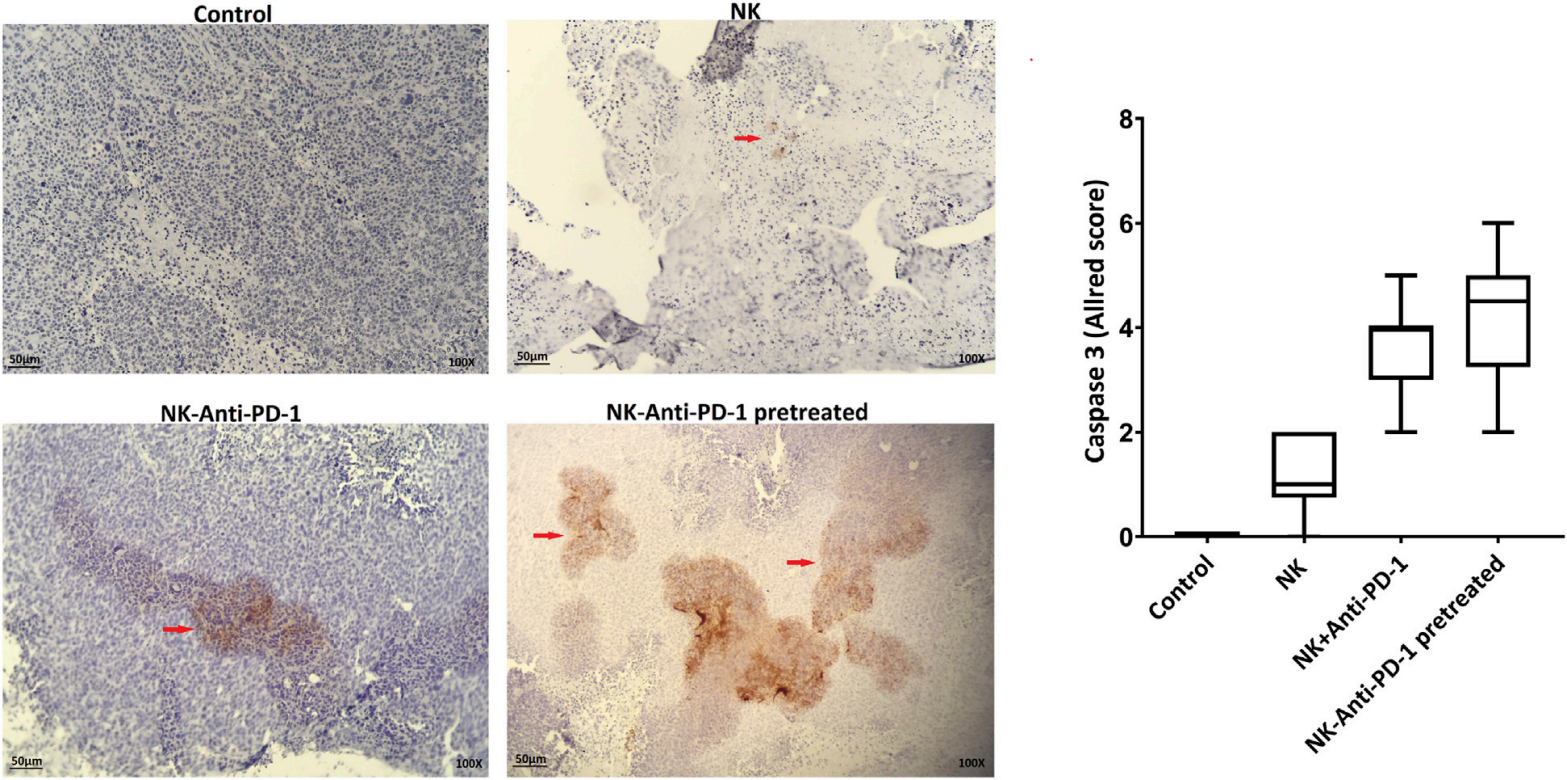
Apoptosis induction by anti-PD-1 blockade NK cells. **(A)** Active caspase-3 was evaluated in the tumor section (red arrow, immunoreactive cells) and boxplots illustrate semi-quantitative analysis based on Allred score. Scale bar: 50 µm, 100X magnification.

The cytotoxicity mechanism of different treated groups *via* apoptosis was evaluated by caspase 3 labeling. Our findings based on Allred score show that NK cells-anti-PD-1 pretreated and NK-Anti-PD-1 groups have intermediate level of caspase 3, while NK cell alone was weakly positive for caspase 3 (Figure 6).

### *In Vivo* Treatment of NK-Anti-PD-1 Increased Lymphocyte Infiltration

**FIGURE 7:**
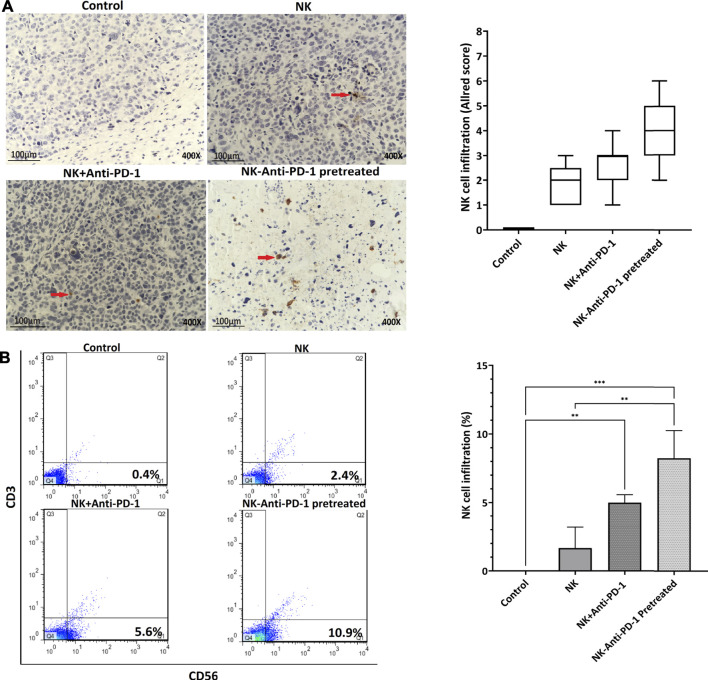
*In vivo* treatment of NK cells increased lymphocyte infiltration. **(A)** Tumor microenvironment infiltrating NK cells were evaluated by IHC with anti-CD56 antibody in tumor masses (red arrow showed immunoreactive cells) and boxplots indicated semi-quantitative analysis based on Allred score. **(B)** Quantification of lymphocyte infiltration performed by whole tumor flow cytometry with anti-CD56 and anti-CD3 antibody, CD56+and CD3-cells showed infiltrating NK cells. Data presented as means ± standard error (M ± SE) for three independent experiments. Statistical significance was determined using one-way ANOVA with *p < 0.05. Scale bar: 100 µm, 400X magnification.

In order to verify the NK cell permeation, human CD56^+^ cells were evaluated. NK-anti-PD-1 pretreated cells were intermediate positive and the other treated groups that received NK cells were weakly positive for CD56 biomarker based on Allred score (Figure 7A). The infiltration percentage of the NK cells, NK + anti-PD-1, and NK-anti-PD-1 pretreated cells groups were 3, 5, and 7%, respectively. Thus, this result indicated that NK-anti-PD-1 pretreated group showed the highest infiltration (*p* < 0.008) (Figure 7B).

## Discussion

The low number and infiltration of NK cells in the tumor area are challenging in GC patients (Li, Zhang et al., 2016; Peng, Zhang et al., 2017). PD-1 receptor as ICIs molecules negatively regulates T and NK cells. Therefore, GC tumors using PD-1 receptors inhibit the patient ‘s immune system. (Lesokhin, Callahan et al., 2015; Liu, Cheng et al., 2017). Accordingly, PD-1 inhibitor has been shown to enhance immunity in clinical trials of non-small cell lung cancer (NSCLC), colorectal cancer, gastric cancer, head, and neck squamous cell carcinoma (Brahmer, Rodríguez-Abreu et al., 2017; Kang, Boku et al., 2017; Overman, McDermott et al., 2017; Tahara, Muro et al., 2018). Recent advances in combination therapy targeting tumor microenvironment or immune checkpoint molecules seem advantageous for NK cell therapy.

In this study, anti-PD-1 antibodies were combined with NK cell-based therapy in gastric cancer xenograft mouse models. As reported in previous studies, a blockade of immune checkpoints can increase the efficiency of NK cell therapy by increasing cytotoxic activity. Bo Yuan Huang et al. and Shevtsov et al. (2019) have shown that PD-1 inhibited the increase in cytotoxic potency of NK cells by up to 10% (Huang, Zhan et al., 2015; Shevtsov, Pitkin et al., 2019). While the present study indicated *ex vivo* expanded IL-2-activated NK cells have therapeutic cytotoxic potential toward MKN-45 cells *in vitro*, anti-PD-1-treated IL-2-activated NK cells improved cytotoxicity of NK cells by 5%.

It has been confirmed that the mechanism underlying tumor recognition by NK cells was mainly through human lymphoid stress surveillance. These cells recognize and kill the cells that express NKG2D ligands (Shafi, Vantourout et al., 2011). NKG2D ligands were downregulated on normal tissues, whereas they were upregulated on the malignant cells (Groh, Bahram et al., 1996). Furthermore, the crucial role of NKG2D-mediated tumor surveillance is confirmed by the rapid elimination of the tumor cells that were transfected with NKG2D ligands by immune cells (Diefenbach, Jensen et al., 2001). In addition to NKG2D, the non-exclusive cytotoxic mechanism of NK cells and other molecules like CD69 might be implicated as a proliferative and cytotoxic marker (Borrego, Robertson et al., 1999). Our results indicated that anti-PD-1 blockade could promote an elevation of NKG2D and CD69 expression levels, and this increase could be modulated by immune-promoting molecules (Marçais, Viel et al., 2013). In confirmation of our results, Dai et al. have shown that anti-PD-1 antibody increases NKG2D and other cytotoxic factors (Dai, Lin et al., 2016), demonstrating the synergistic effect of these therapeutic approaches. Our proof-of-principle study has illustrated the promising results of a combination therapy of *ex vivo* expanded IL-2-activated NK cells and anti-PD-1 antibody. The therapeutic efficacy observed in this experiment is in line with the previously reported effects (Janjigian, Bendell et al., 2018; Patel and Minn 2018). Furthermore, Chen et al. have performed a gastrointestinal (GI) cancer meta-analysis, which showed low responsiveness to ICIs molecules (Chen, Zhang et al., 2019). Therefore, the different cytotoxic improvements in these studies may be due to the different response rates of gastric cancer cells and different E: T ratios in this study.

*In vivo* interventions started at the 100–200 mm^3^ of xenograft tumor volume, equivalent to the advanced stages of human tumors (Alley, Hollingshead et al., 2004). Combination groups improved TGI, leading to proliferative activity reduction linked to better survival rates and increasing apoptotic bodies and fibrotic areas. In accordance with the tumor mitotic counts and apoptosis results, the level of caspase 3 in NK-anti-PD-1 pretreated group was highly increased. Moreover, Yin et al. and Oyer et al. have indicated that cytotoxicity and antitumor efficacy of NK cells recovered when combined with anti-PD-1/PD-L1 blockade (Oyer, Gitto et al., 2018; Yin, Di et al., 2018). On the other hand, J Li et al. have shown that the knockdown of PD-L1 in human gastric cancer cells significantly improved the cytotoxic sensitivity to CIK (cytokine-induced killer cells) therapy (Li, Chen et al., 2017). These results showed that a combination strategy might be an effective and promising approach for GC immunotherapy.

Antitumor responses in immunodeficient mice were accompanied by an infiltration of the tumors; when PD-1 blockade was used, more infiltration induction was observed than that in the other groups. Although NK cell or PD-1 blockade therapies individually elicit an antitumor response, combination therapy is significantly more effective. Konrad Kokowski et al. have shown that the combination of NK cell transfer and radiochemotherapy with second-line PD-1 inhibition improved the overall survival of a patient with NSCLC stage IIIb and induced a massive NKG2D + immune cell infiltration (Kokowski, Stangl et al., 2019). The effect of the anti-PD-1 antibody on lymphocytes expressing the PD-1 receptor can cause this synergistic effect. This combination strategy also showed high NK cell infiltration in preclinical models of lung cancer and glioblastoma (Shevtsov, Pitkin et al., 2019). The moderate response obtained to the combined strategy *in vitro* compared to *in vivo* is probably due to differences in PD-1/PDL-1 expression and other stimuli of this ligand and receptor in 2- and 3-dimensional environments (Terme, Ullrich et al., 2011; Jung 2014; Abiko, Matsumura et al., 2015). Moreover, the mild antitumor effect achieved by monotherapy of NK cells resulted in a delayed tumor progression, which was not significant, that probably required continued therapy at higher doses.

In our study, we used the PD-1 inhibitor for improving adaptive NK cell therapy. However, other ICIs (e.g., anti-LAG3/PD-1, anti-NKG2A, anti–TIM-3 antibodies) have been reported to regulate antitumor functions of NK cells and thereby elevate their cytotoxic activity (André, Denis et al., 2018; Lanuza, Pesini et al., 2020; Zhang, Jiang et al., 2020). Thus, the combination therapy approach of IL-2-activated NK cells with several therapeutic antibodies can decrease exhaustion and enhance the cytotoxicity of NK cell-based immunotherapies.

The use of PD-1 blockade can cause other lymphocytes to accumulate in tumor sites such as cytotoxic T cells; NOG mice do not have immune cells (Shultz, Lyons et al., 2005). Therefore, the presence of other immune cells could not be assessed. However, the absence of an immune system confirms the antitumor function of NK cells, specifically with PD-1 blockade. Furthermore, a better clinical response is presumably observed in the presence of a complete immune system in clinical trials from this therapeutic approach.

## Conclusion

Our results demonstrate the highest response of the combined strategy of NK and anti-PD1 in high T: E *in vitro,* and anti-PD-1 treatment improved proliferation and cytotoxic properties of NK cells. Furthermore, adaptive NK cells therapy with low efficacy in the monotherapy approach could be improved by adding an anti-PD-1 antibody, and the pretreated strategy was more effective against the gastric cancer animal model. Therefore, the combination approach of *ex vivo* expanded IL-2-activated NK cell and PD-1 blockade is promising, and its effectiveness could be evaluated in randomized clinical trials for gastric cancer.

## Data Availability

The original contributions presented in the study are included in the article/supplementary material; further inquiries can be directed to the corresponding author/s.
